# Gabapentin for Pain Control in Burn Patients for Surgical Debridement: Pharmacokinetic Properties Consideration

**DOI:** 10.5812/atr.7068

**Published:** 2012-10-14

**Authors:** Tun Hing Lui, Chi Shan Lam

**Affiliations:** 1 Department of Anaesthesiology and Operating Services, Alice Ho Miu Ling Nethersole Hospital and North District Hospital, Hong Kong, China

**Keywords:** Gabapentin, Burns, Pain

*Dear Editor,*

To the Editor – Rimaz and Colleagues should be congratulated on their report of the effect of Gabapentin on postoperative burn patients (1). There are few points concerning the pharmacokinetic properties of Gabapentin on postoperative burn patients that are worth considering. Firstly, Richardson (2) reminded us to observe pathophysiological changes that follow burn injury before prescribing any drug to a burn patient. During the first 48 h, decreased organ blood supply will reduce clearance of drugs, but the subsequent hypermetabolic phase (48 h after injury) is associated with increased clearance. Variations in levels of acute phase plasma proteins lead to changes in drug binding and free fractions available for end action. The review by Kong and Irwin (3) stated that Gabapentin is not bound to plasma proteins. It is extensively distributed in human tissues and fluid after administration. Gabapentin is not metabolized and does not induce hepatic microsomal enzymes. It is eliminated unchanged in the urine and any unabsorbed drug is excreted in the faeces. Elimination rate constant, plasma clearance, and renal clearance are linearly related to creatinine clearance. Therefore, dose adjustment is necessary in patients with compromised renal function. Secondly, stress ulcer prophylaxis which is commonly prescribed in burn patients could affect renal clearance of Gabapentin. For example, Cimetidine, a H2 receptor blocker, decreases the renal clearance of Gabapentin by 12% when administered concomitantly (3). Thirdly, delirium is a concern in both burn and postoperative patients (4). In Leung’s pilot study, there was significantly less delirium in patients having perioperative Gabapentin (P = 0.045) in the dose of 900 mg started 1 – 2 h before spinal surgery and continued for the first 3 postoperative days. The mechanism of Gabapentin reducing postoperative delirium is unknown. It could possibly be related to its opioid sparing effect (5).

## References

[1] Rimaz S, Emir Alavi C, Sedighinejad A, Tolouie M, Kavosi S, Koochakinejad L (2012). Effect of Gabapentin on Morphine Consumption and Pain after Surgical Debridement of Burn Wounds: A Double-Blind Randomized Clinical Trial Study.. Arch Trauma Res..

[2] Richardson P, Mustard L (2009). The management of pain in the burns unit.. Burns..

[3] Kong VK, Irwin MG (2007). Gabapentin: a multimodal perioperative drug?. Br J Anaesth..

[4] Palmu R, Suominen K, Vuola J, Isometsa E (2010). Mental disorders among acute burn patients.. Burns..

[5] Leung JM, Sands LP, Rico M, Petersen KL, Rowbotham MC, Dahl JB (2006). Pilot clinical trial of gabapentin to decrease postoperative delirium in older patients.. Neurology..

